# Differential neuromuscular adaptations following multi-joint vs single-joint resistance exercise training

**DOI:** 10.1007/s00421-026-06391-7

**Published:** 2026-08-08

**Authors:** Andrea Monte, Vasilios Baltzopoulos, Paola Zamparo, Dave Clarck, Nicola Lucietto, Constantinos N. Maganaris

**Affiliations:** 1 Department of Neurosciences, Biomedicine and Movement Sciences, University of Verona, Via Casorati 43, Verona, Italy; 2 Research Institute for Sport and Exercise Sciences (RISES), Liverpool John Moores University, Liverpool, UK

**Keywords:** Muscle architecture, EMG, Knee extensors, F-L relationship

## Abstract

We combined ultrasound, EMG and dynamometry measurements to establish differences in neuromechanical adaptations following two resistance training programs of equal training volume, one involving a multi-joint exercise and the second a single-joint exercise. Twenty-four participants were divided into a squat group (SQG) and a leg extension group (LEG). Both groups performed ten weeks of training. Before and after the training protocol, muscle architecture of the knee extensors was quantified in vivo using an ultrasound apparatus. The vastus lateralis (VL) torque-fascicle length relationship was also obtained. The maximum isometric torque (Tmax), the maximum EMG activity and the optimal VL fascicle length (L_0_) were determined. A significant increase in 1RM (*P* < 0.001) was observed in both groups post-training. Muscle thickness increased in both groups (*P* = 0.008 and *P* < 0.001 LEG and SQG, respectively), but the increase was greater in SQG than LEG (*P* = 0.031). Both groups showed significant changes in Tmax (*P* = 0.0084 and *P* = 0.0044 for LEG and SQG, respectively) and the improvement in T_max_ was larger (*P* = 0.041) for SQG (+ 14%) than LEG (+ 10%). Pennation angle, fascicle length and VL L_0_ increased post-training in SQG only. A transfer effect of 1RM was observed in both groups: SQG improved the leg extension 1RM by about 16%, whereas LEG increased the squat 1RM by about 9%; SQG showed a larger strength improvement transfer than LEG (*P* = 0.037). Due to the high knee torque required to maintain a given external load, squat exercise training may be more effective than the leg-extension setup used in the present study in eliciting neuromuscular adaptations.

**Supplementary Information:**

The online version contains supplementary material available at https://doi.org/10.1007/s00421-026-06391-7.

## Introduction

esistance training programs (RT) have widely been used to improve sport performance, increase muscle mass and strength, or prevent muscle wasting in response to disuse and ageing. In order to optimize results, the prescription of RT programs should be based on scientific principles that consider the manipulation and combination of several variables, including exercise load, velocity (Wilson et al. 1994), range of motion (Blazevich et al. 2009), intensity and training volume (Blazevich et al. 2003).

A further factor that should be taken into consideration is the movement pattern of the exercises executed during the RT program. Depending on the movement pattern, the muscle or muscle group of interest can be loaded by using multi-joint (MJ) or single-joint (SJ) exercises. SJ exercises involve the application of resistive load distal to the primary joint the muscle crosses and have a rotational movement pattern, (i.e., the resistive load travels over an approximate arc around the joint as the exercise is performed). No other joints contribute to the movement pattern trained. An example of a SJ exercise for the quadriceps muscle is leg extension, typically performed using resistance machines. In MJ exercises, instead, moving the resistive load necessitates the contribution of multiple joints, including the primary joint that the muscle crosses. An example of MJ exercise for the quadriceps muscle is the squat exercise. Here the load is placed at the shoulders and travels over a quasi-linear path, with the hip, knee and ankle joints being involved in moving the resistive load.

The above differences between SJ and MJ exercises are reflected in the nature of training adaptations acquired. As shown by Gentil et al. (2017) and Jones et al. (1989), there are greater improvements in multi-joint movement performance (e.g., walking, running and stand-up test) than in the strength of the muscle group trained (e.g., maximum isometric force of the trained muscle group) in response to MJ compared to SJ exercise training, probably due to the similarity between squat movement and the functional test performed. Moreover, Paoli et al. 2017) observed that for a given total work, MJ exercises were more effective for improving muscle strength compared to SJ exercises. A possible explanation for the above differences is the inferiority of SJ compared to MJ exercise training for improving movement performance due to the SJ exercise targeting one muscle only amongst those contributing to rotating multiple joints in a certain way, dictated by the movement executed. One other explanation might be that MJ exercise training improves muscle activation more than SJ exercise training. Stien et al. (2021) observed that the differences in EMG amplitude between single (e.g., leg extension) and multi-joint exercises (e.g., leg press) appear to be exercise- and muscle-specific. Leg press exercises target several lower-limb extensors, whereas leg extension exercise may better target specific muscles (e.g., the rectus femoris). However, although training-induced increases in quadriceps EMG activity were observed following both leg press and knee-extension training, no clear differences in neuromuscular adaptation between exercise modalities were reported (Stien et al. 2020). Therefore, the extent to which single- and multi-joint exercises differentially influence neural adaptations remains incompletely understood.

Differences in muscle architectural adaptations between the two training modalities should also be considered. A meta-analysis of Gentil et al. (2017) concluded that no differences in muscle cross-sectional area and thickness exist between training modalities, indicating similar gains in muscle strength post-training, although the total training volume was different between training groups in many of the studies reviewed. In contrast, Kassiano et al. (2026) showed that, for a given training intensity, leg extension exercise training induced greater rectus femoris hypertrophy, while squat exercise training promoted greater vastus lateralis hypertrophy, particularly at the distal site. However, again, the total training volume differed between the training groups, as in many studies included in Gentil’s review. Hence, the differences in muscle architectural adaptations between SJ and MJ exercises, when standardised in terms of training volume, remain unclear.

However, exercise modality might affect muscle architectural features other than muscle size alone, which are also important for muscle performance. This is due to differences in the length at which the muscle is loaded between modalities. Consider for example a squat vs. a leg extension exercise. In the squat, the joint torque generated by the knee extensors is maximum at the beginning of the concentric phase, where the quadriceps muscle is elongated compared to its resting length. On the contrary, during leg extension, torque varies throughout the range of motion due to the leg’s position relative to gravity and the shape of the rotating cam (Folland and Morris 2008; da Costa-Machado et al. 2026). These differences could lead to different muscle geometrical adaptations. Indeed, Norkovic et al. (2014) tested the effects of 6-weeks of isometric strength training of the knee extensor muscles when the quadriceps was trained at a long or short length, dictated by knee angle configuration. Participants who trained at shorter muscle lengths showed significant increases in vastus lateralis fascicle length in the middle portion of the muscle. In contrast, those who trained at longer lengths exhibited significant increases in vastus lateralis fascicle length in the distal region of the muscle. Post-training increases in fascicle length in vivo are considered to represent in-series sarcomerogenesis, with implications for muscle excursion range and the fascicle length at which maximum contractile force (or torque) is produced (Reeves et al. 2003; Blazevich et al. 2009).

To better understand how muscle structural, neural and functional adaptations may differ between SJ and MJ exercise training programs, in this study, we investigated changes in muscle architecture, muscle activation, strength and torque-fascicle length (T-L) characteristics of the knee extensor muscles after a ten-week training period of squats (Squat Group, SQG) or leg extensions (Leg Extension Group, LEG), standardized for exercise intensity, number of repetitions and range of movement. We hypothesised similar muscle size (e.g., pennation angle and muscle thickness), maximum EMG activity and strength gains between the SQG and LEG post-training. However, due to the differences between exercises in the length at which the muscle is loaded, we expected an increase in fascicle length in SQG only, impacting their T-L relationship.

## Methods

### Participants

Twenty-four healthy untrained males (age: 24.7±2.4 years; body mass: 69.3±3.3 kg; stature: 1.73±3.4 m) participated in the study. All participants were moderately active (sport science students), with no previous involvement in strength training; they were free from any type of injuries, received written and oral instructions before the study and gave their written informed consent to the experimental procedure. The experimental protocol was approved by the Ethical Committee of Liverpool John Moores University (protocol number: 18/SPS/027) and was performed in accordance with the Helsinki Declaration.

### Experimental procedure

**Fig. 1:**
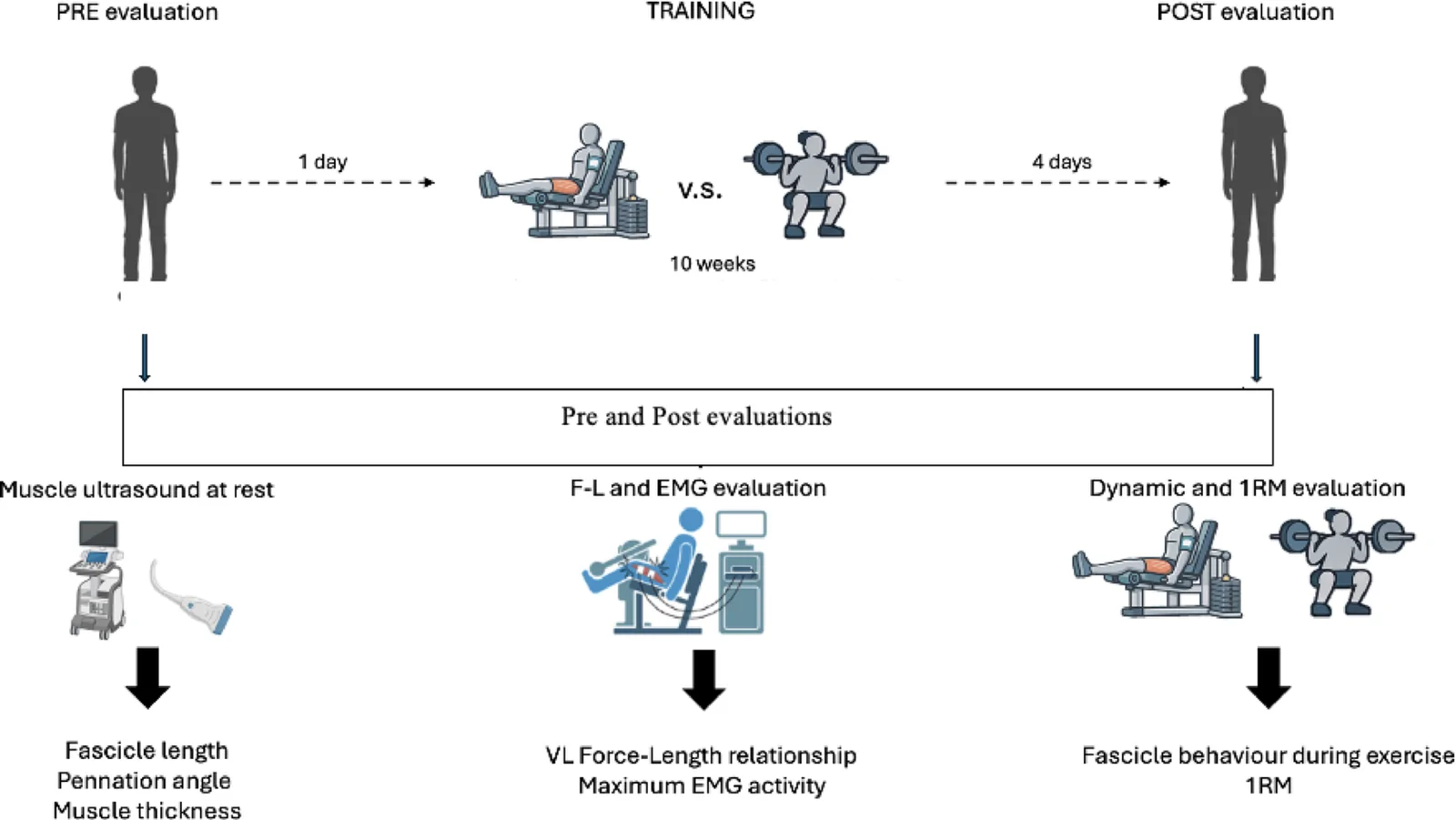
Flow chart with timeline, experimental tasks and principal outcomes

A flow chart with the experimental procedures is depicted in Fig. 1. The participants visited the laboratory on two different occasions for the pre- and post-tests. One and four days of rest were interposed between the pre-test and the first training session, and between the last training session and the post-test, respectively. During the pre- and post-tests the participants underwent the following assessments: Ultrasound scanning at rest to obtain the muscle geometrical characteristics of quadricep muscles, a series of maximum voluntary contractions to reconstruct the vastus lateralis F-L relationship and a series of sub-maximal and maximal dynamic contraction to quantify the fascicle behaviour and the 1RM of the investigated exercises.

### Training protocol

All participants performed three resistance training sessions in the first week to get familiarised with the exercise technique and to learn how to control the range of movement. Participants were then allocated randomly into SQG or LEG for a ten-week training intervention, two times per week. A third session was used to re-evaluate the 1RM for the week after. All participants were tested before and after the interventions were completed.

The training protocol is reported in detail in the supplementary materials. SQG performed barbell back squats and LEG performed leg extensions (model MED; Technogym; Italy). Both legs were trained and the range of knee joint movement was between 0° (full knee extension) and 90°, for the two exercises. Note that the leg extension machine used in this study has a specific cam configuration modifying the resistance in a way that it reaches its maximum value at 0° knee joint angle. The descending phase (eccentric) of both exercises was performed in approximately 2 s whereas the ascending (concentric) phase was performed as fast as possible (to standardise for maximum intent). One rest day separated the two training sessions, and the third session (testing day) was used to re-evaluate the 1RM and adjust exercise intensity.

The training volume (number of repetitions, intensity as % of 1RM, and range of knee joint movement – see below) were standardised for the two groups. The periodisation was characterized by a “linear strategy” where the number of repetitions decreased (from 86 to 27 repetitions from the first to the last week, respectively) and the intensity increased (from 65 to 85% of 1RM from the first to the last week, respectively as a function of the training weeks (see supplementary materials). This progression-protocol design enabled comparing neuromuscular and architectural adaptations while controlling repetition number, relative intensity and range of motion to standardize for total training volume between the two exercises.

### Pre- and post-training measurements

#### Resting muscle architecture

The participants remained in a supine position for the measurements of thickness, pennation angle and fascicle length in the quadriceps muscle. A water-soluble gel was applied to the transducer to aid acoustic coupling and to reduce muscle deformation.

Geometrical characteristics of vastus lateralis (VL), rectus femoris (RF) and vastus medialis (VM) were measured at a standardized distance of thigh length, measured from the knee centre of rotation to the greater trochanter (VL, 50%; RF, 60%; VM, 20%) with an ultrasound apparatus (Healthcare Logiq S7pro, 6 cm and 8 MHz, linear-array probe). The transducer was placed parallel to the specific muscle to observe a longitudinal image with the midpoint of the probe placed longitudinally at the above positions. The transducer was aligned in the fascicle plane with respect to the superficial and deep aponeurosis, to obtain a clear image of the muscle fascicle. The distance between subcutaneous adipose tissue interface and inter-muscular interface was taken as muscle thickness. Pennation angle was measured as the intersection between fascicles and the deep tendon aponeurosis, whereas the fascicle length between the two aponeuroses was measured manually, taking into account any curvature present.

#### Measurements during maximum voluntary contractions (MVCs)

The participants were secured to a customised isometric chair, fixed with a trunk and pelvic strap, with their arms crossed in front of their chest. The F-L relationship was calculated from MVC data at various knee angles. Eight MVCs of the right leg were performed from 20° to 90° of knee joint angle. The participants were instructed to push as hard as possible for 4 s (and received strong verbal encouragement to do so), with 2 min of recovery between MVCs. An ankle strap was placed in line with the lateral malleolus, in series with an aluminium strain gauge (System Pese, Italy; linear response 1500 N) that was perpendicular to the tibia. The distance between the knee centre of rotation defined as the most prominent point on the femoral epicondyle and the strain gauge position represents the external force moment arm about the knee joint. The force signal was amplified (x1000) and sampled at 1500 Hz using an external A/D converter (PowerLab, ADInstruments) and recorded with a personal PC using LabChart 7. For motivation purposes to achieve maximum activation, real-time biofeedback was provided on the PC screen. The product of maximal external force and external moment arm was the maximal knee joint torque produced.

During the MVC, VL fascicle length changes were captured by means of an ultrasound apparatus (as described previously). The probe was attached to the skin using a custom-made involucrum approximately at 50% of the femur length and aligned with respect to the superficial and deeper aponeurosis, in order to have a clear image of the perimysial connective intramuscular tissue, that it is indicative of the muscle fibre structure. Based on these data the torque-VL fascicle length relationship was calculated, as described in detail below.

Finally, the EMG activity of the VL and biceps femoris (BF), were recorded during the knee extensor MVCs to account for potential antagonist muscle co-activation effects. Two bipolar Ag-AgCl electrodes were placed in the central region of the muscles after skin preparation (shaving, gentle abrasion and cleaning with an alcohol-based tissue pad. The raw EMG data were recorded at 1500 Hz with the same software used for the force signal.

All instrumentation was synchronised with an external manual trigger (5 V).

#### Dynamic contractions and 1RM evaluation

For each participant, 1RM was assessed not only for the exercise they were allocated to, but also for the other exercise, to evaluate transferability of muscle strength between exercise protocols. The evaluation of 1RM was performed in the morning for one exercise and in the afternoon for the second exercise. The exercise evaluation order was randomised.

After 5 min of warm-up (cycling at a self-selected intensity), the participants performed a series of sub-maximal loading exercises at different loads. One-RM testing for both the squat and leg-extension exercises was performed using a standardized incremental ramping protocol adapted from NSCA recommendations. The load progression was individualised based on the participant’s performance, with larger increments during the initial stages and smaller adjustments as near-maximal loads were approached. Between attempts, participants were allowed 2–4 min of recovery to minimise fatigue. One-RM was defined as the highest external load that could be lifted through the prescribed range of motion without external assistance. Typically, maximal load was achieved within three to five testing attempts. For both exercises, failure was defined as the inability to complete the concentric phase through the predefined knee joint range of motion.

After the 1RM test, each participant performed three contractions at five sub-maximal loads: 15, 30, 45, 60 and 75% of the 1RM of squat or leg extension exercise. During these contractions, 3D marker trajectories were recorded using 8 Vicon cameras (MX-20, Oxford Metrics, UK) sampling at 200 Hz. The marker-set used in this protocol was the same used by Monte et al. (2020). Furthermore, during the squat exercise, ground reaction forces were recorded using two individual force plates, one for each leg, (Kistler 600 × 400) at 1500 Hz; while a 2D camera (100 Hz) was utilised to record the leg extension kinematics to reconstruct the subject position in respect to the machine.

In addition, a B-mode ultrasound described earlier was used to record images at 60 Hz with a depth and width of 6 cm, by fixing the scanning probe on the skin using a customised holder. Fascicle displacement of VL was calculated by an automatic tracking software, as was also the case for the isometric contractions utilised to investigate torque-fascicle length characteristics (see data analysis).

The EMG activity of VL and of BF were also recorded at 1500 Hz during all submaximal and maximal dynamic contractions. The location of the electrodes was the same in the dynamic and isometric contractions, based on markings made on the skin during subject preparation.

### Data analysis

#### Muscle architecture

Three ultrasound images were recorded and analysed for each geometrical feature and muscle with a free software (Image J, https://imagej.nih.gov/ij/) by the same operator. The mean value was utilized for further analysis. To test the reliability of the ultrasound procedure the mean coefficients of variation (mean among muscles) was calculated for each parameter: 2.38% (range:1.93–2.54%) for muscle thickness, 2.45% (range: 2.28–2.71%) for pennation angle and 2.61% (range: 2.33–2.96%) for fascicle length.

#### Isometric contractions (MVCs)

Force-time signals were filtered with a low-pass Butterworth filter (cut-off frequency: 30 Hz). After correcting for the antagonist BF negative contribution during the MVC tests (Kellis and Baltzopoulos 1996, 1998; Maganaris et al. 1998), the corresponding value of maximum external force (N) in each MVC was multiplied by external moment arm (m) to calculate the net maximum knee joint torque (Nm).

To reconstruct the torque–VL fascicle length (T–L) relationship, the peak torque value recorded at each knee joint angle was identified and the corresponding fascicle length was manually measured. Torque and fascicle length data obtained across knee angles were then fitted with a second-order polynomial equation (Bohm et al. 2018; Monte et al. 2020), from which maximal isometric torque (Tmax) and the optimal fascicle length (Lo; i.e., the fascicle length at which Tmax occurred) were determined for each subject.

The EMG activity during the MVCs was used to calculate the RMS value for each contraction in all the analysed muscles (with a time window of 1 s after the plateau of the force signal). The highest RMS values determined during the isometric MVCs was then used to normalise the RMS values attained during the dynamic contractions (which was thus expressed as %MVC). The EMG-time signal of all the investigated muscle was filtered with a fourth order band-pass Butterworth filter (band-pass frequency: range 20–450 Hz).

#### Dynamic contractions (squat and leg extension contractions)

The marker trajectories were filtered with a forward and reverse second order Butterworth filter (cut-off frequency: 20 Hz). These data were utilized to calculate the knee angle range of motion and to identify the contraction phases (eccentric and concentric).

Fascicle lengths for VL were calculated from the ultrasound videos by a validated automatic tracking algorithm (Gillett et al. 2013). At the end of the auto-tracking, every frame was visually examined to check the algorithm accuracy. Whenever the fascicle length measurement was deemed inaccurate, the two points defining the origin and insertion of muscle fascicles were manually repositioned.

The EMG-time signal was filtered, as previously described for the isometric contractions; in this case, the RMS was calculated from the beginning to the end of the concentric phase. The RMS values were then normalised for the maximum RMS values reached during the isometric contractions.

The values of 1RM were expressed in kg and used to evaluate the differences in muscle strength between training groups.

Finally, the knee torque was calculated by combining kinematic and kinetic data. For the squat, the knee joint torque was calculated through inverse dynamics. For the leg extension, the knee torque was calculated as the product of weight utilised in a specific contraction multiplied by *g* (9.81 m/s^2^) and by the external force moment arm length obtained from the 2D video analysis, which was taken as the shortest or perpendicular distance between the estimated knee joint centre of rotation, which was aligned with the centre of rotation of the leg extension apparatus, and the direction of the cable at the level of malleoli that carried the external load lifted.

### Statistics

Normality distribution was assessed by means of Shapiro-Wilk in combination with the Q-Q plots. The Homogeneity of Variance-Covariance matrices was evaluated and confirmed.

Changes in muscle architecture, isometric and dynamic EMG activity, T-L parameters (T_max_ and Lo), fascicle behaviour and 1RM were assessed using repeated measures MANOVA (group: between-subject; Pre-Post: within-subject). When a significant difference was observed a Bonferroni’s post hoc analysis was used. An alpha level of *P* < 0.05 was set as the criterion for statistical significance. SPSS (v.24) was used for the analysis. All data are reported as mean±standard deviation.

## Results

### 1RM

Both groups showed a significant increase of 1RM after the training protocol (*P* < 0.001) (SQG: 130±10 kg and 165±15; LEG: 52.5±5 kg and 62.5±5 kg for pre and post-test, respectively), which corresponds to an improvement of 27% and 18% for SQG and LEG, respectively. The improvement in 1RM was different in the two groups (*P* = 0.0078).

### Muscle architecture and optimal length

Data of muscle thickness, pennation angle, fascicle length (for all muscles) and optimal fascicle length (for VL only) are reported in Table 1. On average, for all muscles, muscle thickness increased in both groups (*P* = 0.0083 and *P* < 0.001 for LEG and SQG, respectively) but the increase was greater in SQG than LEG (15% and 4%, respectively). On the contrary, pennation angle and fascicle length increased post-training in SQG only (*P* = 0.0066 and *P* < 0.001 for pennation angle and fascicle length, respectively), with no changes in LEG.

### Knee joint torque

**Fig. 2:**
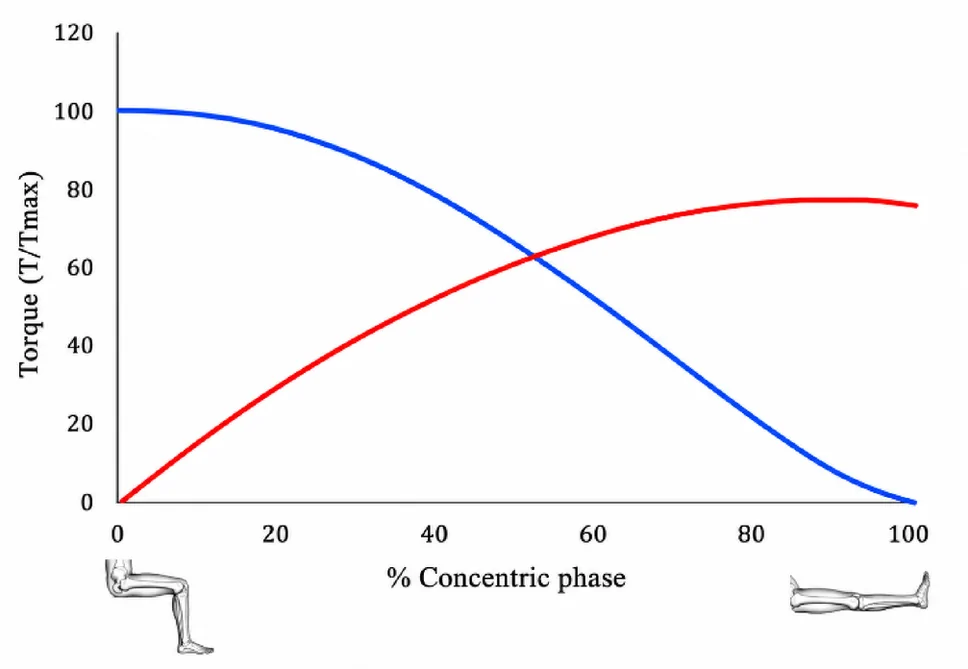
Representative average values of knee joint torque exerted during squat (blue line) and leg extension (red line) at 75% of the 1RM. Squat and leg extension data are normalised for the maximum isometric torque. Similar behaviour was observed for all the external sub-maximal intensities

Data of knee joint torque during the squat exercise (SQG) and leg extension exercise (LEG) as a function of range of motion at 75% of 1RM are shown in Fig. 2. Similar results were also obtained at 15, 30 and 60% of 1RM. There were differences in both curve profile and magnitude of torque between the two exercises. For the squat exercise, the knee joint torque was maximum at the beginning of the concentric phase and minimum at the end. For the leg extension exercise, instead, the knee torque was maximum at the end of the concentric phase and minimum at the beginning. Consequently, when the knee extensor muscles were elongated, the torque was maximum for the squat and minimum for the leg extension. On the contrary, at the shorter muscle length, the torque was lower for the squat and higher for the leg extension.

In terms of normalised torque magnitude, during the concentric phase of the squat, the knee extensors generated on average 26, 31, 39, 46 and 56% of the maximum voluntary torque to lift a load that corresponded to 15, 30, 45, 60 and 75% of the 1RM. For the leg-extension exercise at the same percentages of the 1RM, the knee extensors generated 21, 25, 33, 42 and 50% of torque exerted during the isometric knee extension MVC. Consequently, the torque exerted by the knee extensor muscles was larger for the squat compared to the leg extension (*P* = 0.0011).

### Isometric and dynamic EMG activity

The maximum EMG activity during MVCs increased post-training in both LEG and SQG (%difference from pre to post-training: +15.2%, *P* = 0.0067 and + 32.7%, *P* < 0.001 for LEG and SQG, respectively) with a significant difference found between groups (*P* = 0.0054).

**Fig. 3:**
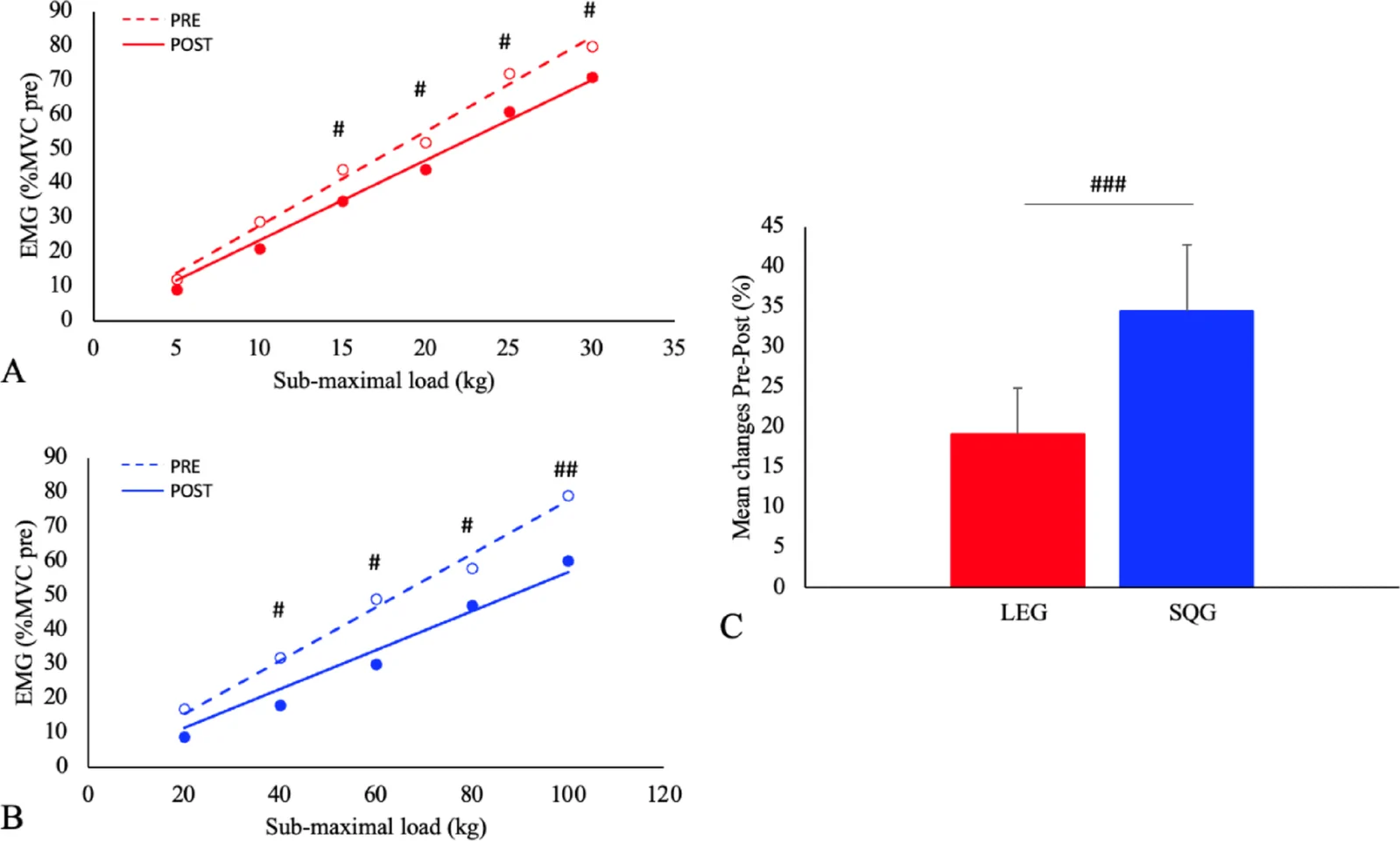
EMG activity during sub-maximal squat and leg extension loads in SQG and LEG are shown in A and B, respectively. Values are shown relative to MVC pre-training. Dashed and continuous lines refer to pre and post-training, respectively. C: mean values of EMG changes obtained in both training groups. Red and blue data refer to the leg extension and squat training group, respectively #*P* < 0.05; ##*P* < 0.01; ###*P* < 0.001

The EMG activity during the sub-maximal loads of the dynamic exercise is reported in Figs. 3A (LEG) and 2B (SQG). Post-hoc tests are reported in Fig. 3. The mean values of % changes in EMG activity for both groups are reported in Fig. 3C. SQG showed a larger reduction in EMG than LEG post-training (*P* = 0.028).

### Torque-fascicle length relationship

**Fig. 4:**
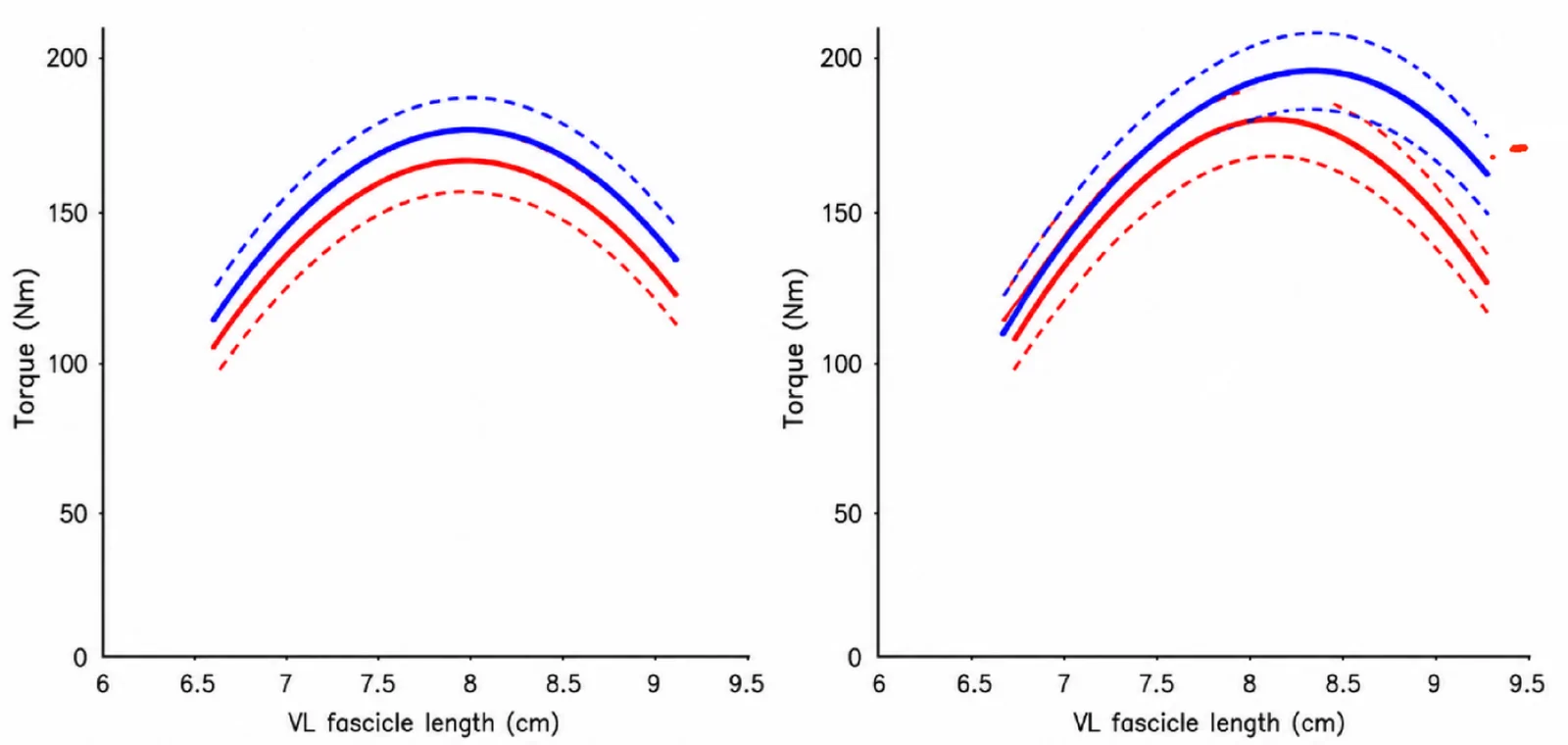
Torque-fascicle length relationship for the Vastus Lateralis for LEG (left panel) and SQG (right panel) pre (red lines) and post-training (blue lines). Continuous lines are the mean values, while the dashed lines refer to the standard deviation. Note the upward shift of the curve for both groups post- training and the concomitant rightward shift of Lo for SQG only

The T-L relationships pre and post-training are reported in Fig. 4. Both groups showed significant changes in Tmax: 167±15 and 174±19 Nm before and 184±19 and 200±21 Nm after the training program for LEG (*P* = 0.0084) and SQG (*P* = 0.0044), respectively, corresponding to an improvement of + 10% and + 14%, respectively. The improvement in T_max_ was larger for SQG than LEG (*P* = 0.041). There was a significant increase in VL Lo post-training in SQG, resulting in a rightward shift on the T-L relationship (*P* = 0.021). There were no changes in VL Lo post-training in LEG.

### Fascicle behaviour during dynamic exercise

**Fig. 5:**
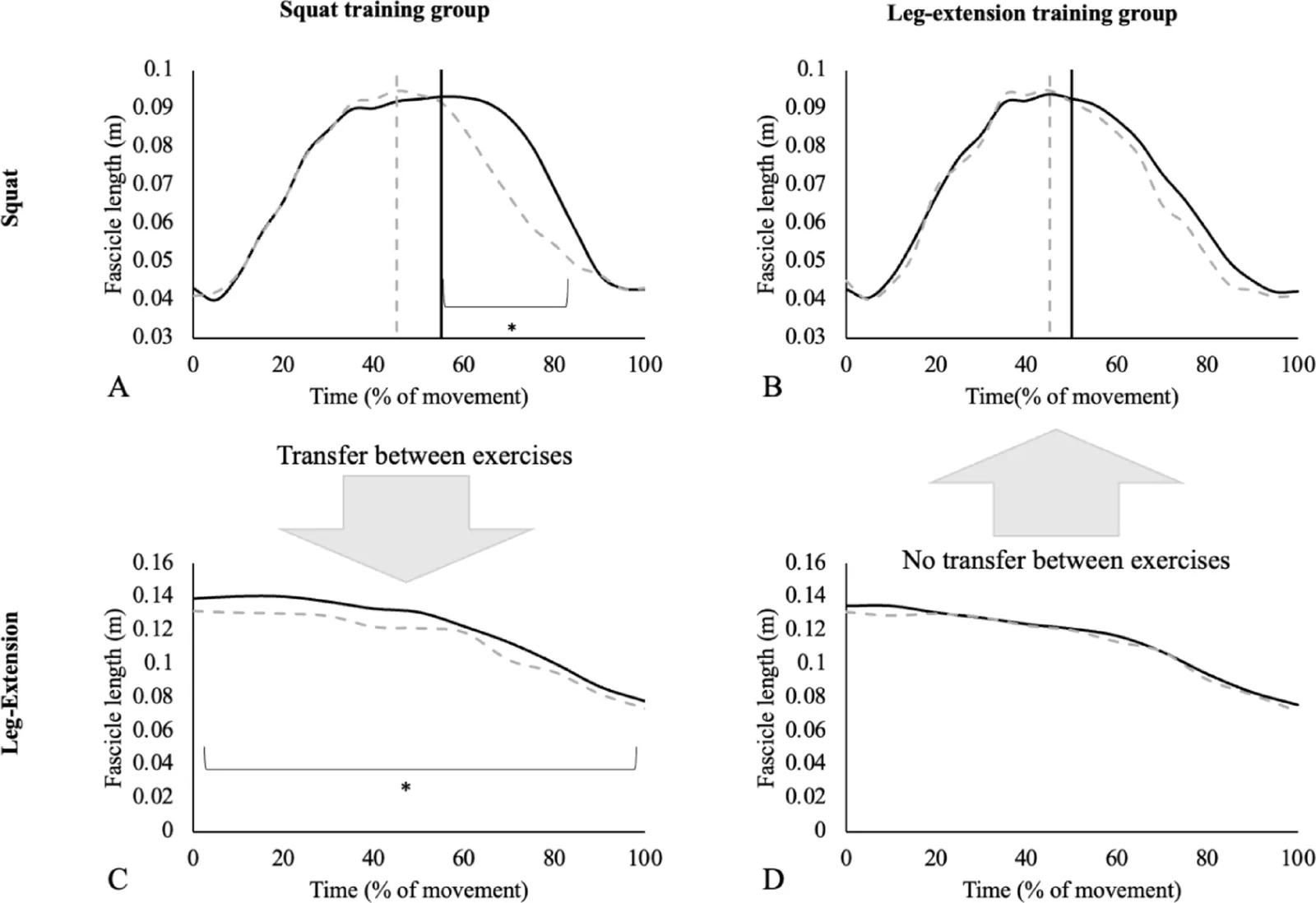
Example of vastus lateralis fascicles behaviour during squat and leg extension at 60 kg and 25 kg. Each panel refer to the fascicle behaviour pre (dashed) and post (continuous) training. Right and left panels refer to the SQG and the LEG training groups, respectively; who performed the squat (upper panel) and the leg extension (lower panel). A transfer effect is evident for SQG, while this was not the case for LEG. *P < 0.05

A representative data set of VL fascicle behaviour during squat and leg extension exercises is shown in Fig. 5 for both SQG and LEG (25 kg for LEG and at 60 kg for SQG). Fascicle length showed significant differences after training in SQG at all sub-maximal loads, except for the lowest condition (20 kg) (see Table 2). Particularly, the mean values of fascicle length during the concentric phase were larger compared to pre-test (*P* = 0.032). No significant differences were observed for LEG at all sub-maximal loads.

**Table 1:** Pre- and post-training values of muscle architecture and optimal length in both training groups

SQG													
		Thickness (cm)			Pennation angle (°)			Fascicle length (cm)			Lo (cm)		
VL	Pre	2.39 ± 0.82			15.60 ± 4.66			8.93 ± 1.55			8.12 ± 1.15		
	Post	2.82 ± 0.79^**^^			17.23 ± 4.89^*^^			9.47 ± 1.49^**^^			8.58 ± 1.09^**^^		
RF	Pre	2.89 ± 0.74			23.30 ± 7.39			7.47 ± 1.21					
	Post	3.12 ± 0.81^*^^			24.51 ± 7.61^*^^			7.88 ± 1.33^**^^					
VM	Pre	3.36 ± 0.96			20.70 ± 7.12			9.70 ± 1.89					
	Post	3.87 ± 1.01^*^^			22.31 ± 8.02^*^^			10.07 ± 1.85^**^^					

Leg extension group													
		Thickness (cm)			Pennation angle (°)			Fascicle length (cm)			Lo (cm)		
VL	Pre	2.26 ± 0.78			15.98 ± 3.75			8.21 ± 1.45			8.07 ± 1.25		
	Post	2.39 ± 0.74^*^^			15.96 ± 4.89^^^			8.49 ± 1.51^^^			8.11 ± 1.21^^^		
RF	Pre	2.73 ± 0.80			22.11 ± 7.01			7.25 ± 1.33					
	Post	2.82 ± 0.93^*^^			21.94 ± 7.61^^^			7.58 ± 1.38^^^					
VM	Pre	3.27 ± 0.88			18.98 ± 6.73			10.05 ± 1.78					
	Post	3.40 ± 0.98^*^^			18.87 ± 8.02^^^			10.32 ± 1.73^^^					

VL: vastus lateralis; RF: rectus femoris; VL: vastus medialis

Significant difference between pre and post training period (*P < 0.05; **P < 0.01)

Significant difference between groups (^)

**Table 2:** Pre and Post mean values of fascicle length during the concentric phase of both exercises in squat and leg extension training groups

Squat group						
Load (kg)	PRE			POST		
	Fascicle length (cm)			Fascicle length (cm)		
20	6.91 ± 0.20			6.99 ± 0.20		
40	6.86 ± 0.22			6.91 ± 0.21^*^		
60	6.67 ± 0.18			6.72 ± 0.17^*^		
80	6.55 ± 0.16			6.62 ± 0.17^*^		
100	6.47 ± 0.15			6.53 ± 0.15^*^		

Leg extension group						
Load (kg)	PRE			POST		
	Fascicle length (cm)			Fascicle length (cm)		
5	8.54 ± 0.22			8.49 ± 0.21		
15	8.45 ± 0.25			8.41 ± 0.22		
25	8.31 ± 0.24			8.25 ± 0.21		
35	8.29 ± 0.21			8.21 ± 0.19		
45	8.18 ± 0.17			8.15 ± 0.18		

Significant difference between pre and post training period (*P < 0.05)

### Transfer between exercises

A transfer effect of 1RM was observed in both groups: SQG improved the leg extension 1RM by about 16% (from 50±5 to 59±5 kg), whereas LEG increased the squat 1RM by about 9% (from 127.5±10 to 140 kg); SQG showed a larger strength improvement transfer than LEG (*P* = 0.025).

**Fig. 6:**
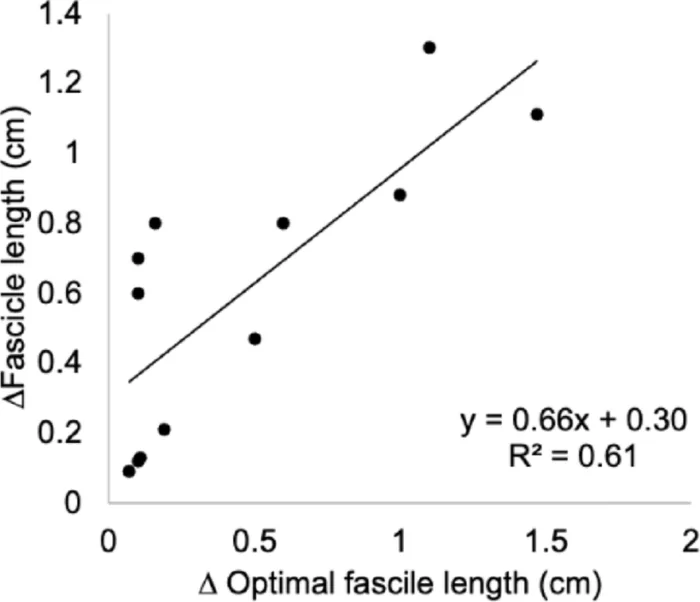
Relationship between changes in resting fascicle length pre-training to post-training and corresponding changes in Lo based on the T-L relationship in SQG (see Fig. 6). The greater the fascicle length increase post-training, the greater the Lo (i.e., rightward sift of Lo in T-L relationship)

In addition, SQG operated at longer fascicle lengths during the leg extension exercise at all the investigated sub-maximal loads (*P* = 0.028). No such effect was observed for LEG (Fig. 6).

## Discussion

This study aimed to investigate neuromuscular changes following ten-weeks of resistance training using squats versus leg extensions, standardized for intensity, repetitions and range of knee joint movement. In contrast to our hypothesis, we showed superior adaptations in SQG compared with LEG. Although muscle hypertrophy occurred in both groups as indicated by the increases in muscle thickness post-training, the increase was greater in SQG than LEG. In addition, pennation angle and fascicle length increased post-training in SQG only. The above muscle architecture adaptations led to a more pronounced increase in Tmax in the T-L relationship in SQG than LEG, and importantly, a rightward shift of Lo in the SQG only. Finally, we observed a greater 1RM transfer effect from SQG to LEG.

The effects of strength training on muscle architecture are well-reported in the literature (Narici et al. 1996; Reeves et al. 2003; Blazevich et al. 2003; Franchi et al. 2014, 2017). The novelty in our study is the finding that muscle architecture adaptations were shown to be affected by exercise modality. SQG exhibited significant increases in all the investigated geometrical variables, while LEG showed a significant increase in muscle thickness only, and this increase was smaller than in SQG. Accordingly, Chilibeck et al. (1998) reported muscular adaptations occurring earlier with SJ in the short term (4 weeks) compared to MJ, but superior positive adaptations in MJ after 8 weeks. In contrast, Gentil et al. (2017) concluded that the adaptations in muscle size and strength are similar post-training in SJ compared to MJ. However, it must be pointed out that the studies included in Gentil’s review involved upper limb or lumbar extensors exercises, which could not be compared to our exercises due to several differences in biomechanical characteristics of the muscles and joints involved. Kassiano et al. (2026) reported greater rectus femoris hypertrophy following leg extension training compared with squat training, whereas squat training elicited superior hypertrophy of the vastus lateralis. However, in their study total training volume was higher in the leg-extension group, which may have contributed to the greater hypertrophic response observed in the rectus femoris. In the present study, although the training program was primarily designed to improve muscle strength rather than maximize muscle hypertrophy, we observed increases in quadriceps muscle thickness of approximately 14% and 5% in the SQG and LEG groups, respectively. These values are consistent with the magnitude of hypertrophic adaptations previously reported following resistance training in untrained individuals (Soares et al. 2024), suggesting that the strength gains we report here were accounted for partly by increases in muscle size, particularly in the SQG.

The differences between exercises reported in our study could be explained by differences in training load and in the torque-angle profile throughout the range of motion. Although we standardised the training volume between the two protocols, our data suggest that for a given relative external load (1RM %), the knee torque is higher for SQ compared to LEG, increasing the final training load (i.e., the product of volume and intensity) for the SQ group. This could result in a more pronounced adaptation in muscle size and pennation angle, which facilitates the packing of more contractile material in a given muscle volume. Moreover, the length at which the contracting knee extensor muscles produce maximum force differs substantially between the two exercises. Many studies suggest a slight only reduction in the patellar tendon moment from full knee extension to 90 degrees of flexion (Tsaopoulos et al. 2006), so the torque data shown in Fig. 1 would deviate only slightly from the corresponding contractile force data. During the squat exercise, the muscular load is maximal at the beginning of the concentric phase, where the muscle is elongated. On the contrary, the maximum muscle load in the leg extension is exerted at the shortest fascicle length. From a physiological point of view, the mechanosensitive signalling mechanisms would be activated when the muscle contracts at a longer length than its resting length (Butterfield and Herzog 2006). Moreover, Ca^2^-dependent signalling cascade is only augmented when the muscle is stretched, as intracellular calcium accumulation occurs through stretch-activated ion channel activation (Butterfield and Best 2009; Butterfield 2010). In addition, metabolic stress as a driver for muscle protein synthesis would be more pronounced when the muscle is mechanically loaded at longer lengths (Lieber et al. 2017). Indeed, recent reviews and meta-analyses have reported greater hypertrophy and, in some cases, larger increases in fascicle length following resistance training at longer muscle lengths (Androulakis Korakakis et al. 2024; Wolf et al. 2025). These characteristics indicate that the superior effectiveness of squats compared to knee extensions for inducing muscle hypertrophy may not be driven only by the higher contractile forces exerted, but also by the longer lengths at which the muscles contract to generate these forces during the squat. It must be pointed out that these considerations are specific to a leg-extension exercise performed using a machine with the torque profile reported in Fig. 2. Different cam geometries can substantially alter the torque-angle relationship imposed on the quadriceps throughout the range of motion (Folland and Morris 2008; da Costa-Machado et al. 2026). Indeed, Folland and Morris (2008) demonstrated that the torque-angle profiles of commercially available leg-extension machines were highly variable and generally flatter (i.e., less curvilinear) than the human knee extensor torque-angle relationship. Across the different machines, torque changed by only + 2.5 to + 22.2% over the ascending limb and by + 37.6 to − 20.5% over the descending limb of the torque-angle relationship, indicating that most machines do not accurately reproduce the physiological variation in knee extensor torque capability throughout the range of motion. Therefore, the current neuromechanical adaptations may not reflect the implementation of strength training programs using leg extension machines with an accommodating resistance profile dissimilar to ours.

We found that SQG had higher EMG improvements during isometric contraction, which in turn allowed them to generate a given sub-maximal torque with a lower EMG compared to LEG (Fig. 2). This adaptation indicates that the quadriceps muscle may be more active during a squat than a leg extension exercise at similar relative intensity levels. We did not evaluate the VL EMG activity during training in the two groups, but Signorile et al. (1994) did so and reported that, indeed, for a given 1RM percentage, the VL EMG was higher during the squats than the knee extensions. The lower EMG activity required to generate a given submaximal torque following squat training suggests improved neuromuscular efficiency, enabling a similar mechanical output with reduced active muscle volume. This adaptation may be beneficial during functional tasks and repeated muscular efforts, where the ability to produce force with lower relative activation could improve movement economy and fatigue resistance.

The more pronounced hypertrophic adaptation and EMG improvement post-training in SQG compared to LEG is consistent with the higher increases in Tmax in SQG than LEG (Fig. 3). Importantly, we also observed a rightward shift of the T-L curve for the SQ group only. Significant shifts toward longer muscle lengths have been shown previously after periods of eccentric knee extension training (Rabita et al. 2000; Blazevich et al. 2007) and could be explained by increases in fascicle length, which infer in-series sarcomerogenesis as an adaptation to loading. An increase in fascicle length has typically been reported as an adaptive response to eccentric training (Franchi et al. 2014, 2017), but here we established a similar effect in SQG. It is unlikely that the increase in resting fascicle length in SQG is an adaptation to the eccentric phase of the squat exercise, as the eccentric phase of the leg extension exercise was equally emphasized, but there were no changes in LEG resting fascicle length. More likely, the application of eccentric loading at long fascicle lengths, a characteristic of squat movement in the lowering phase of the exercise (see Fig. 1), may be a requirement for fascicle length and Lo alterations post-training. The link between changes in resting fascicle length and Lo is further explored in Fig. 4, where we have correlated post-training changes in VL resting fascicle length with the corresponding changes in Lo in SQG and observed that approximately 60% of the changes in Lo can be explained by changes in resting fascicle length. One other mechanism that could explain the remaining variability in this correlation is a training-induced change in the stiffness of the in-series tendon. An increase in the stiffness of the patellar tendon (Reeves et al. 2003), would affect the operating length of the quadriceps muscle fascicles, as the degree of fascicle shortening induced by contraction is dictated partially by the elongation of the tendon (MacDougall et al. 2020; Monte 2020). In this regard, the amount of quadriceps muscle fascicle shortening could be partially attenuated by the increased patellar tendon stiffness, thus making the muscle operate at longer lengths. It may, therefore, be the case that increases in tendon stiffness have accentuated the post-training right shift of the F-L relationship caused by the increases in resting fascicle length.

We showed that there was a greater 1RM transfer effect for SQG. This finding may be linked with the superior hypertrophic adaptations (larger increase in muscle thickness and pennation angle) in SQG, but also the rightward shift of Lo in this group. Our results are in agreement with the findings of Paoli et al. (Paoli et al. 2017) of greater muscle strength transfer improvements for a squat training group compared to a leg extension training group. Similar results were reported by (Stien et al. 2020), who studied transfer between leg press (MJ exercise) and luteal kick back + knee extension (SJ exercises) and showed increases in 1RM for all the investigated exercises, but greater strength transfer effects for the leg press group only. Collectively, all these data show than MJ exercises may be more effective than SJ for muscle strength improvement transfer. One other possible explanation for the greater transfer effect observed in SQG is that multi-joint exercises require a higher degree of intermuscular coordination and stabilization than single-joint exercises. Successful execution of a squat depends not only on the force-generating capacity of the knee extensors, but also on the coordinated contribution of the hip extensors and trunk musculature to stabilize the body. Consequently, squat training may induce neural adaptations that are specific to complex multi-segment movements, including improvements in intermuscular coordination and motor control (Behm and Sale 1993; Carroll et al. 2001). These adaptations may transfer to simpler single-joint tasks, whereas the neural adaptations elicited by leg extension training are likely more task-specific and therefore less transferable to a complex movement such as the squat.

### Methodological considerations and limitations

In our study, we used a leg extension device with the resistance profile shown in Fig. 2. As noted above, resistance profiles can vary across strength machine brands and models, which may limit the generalisability of the present results. It should also be noted that, as in many other studies, we measured muscle architecture from ultrasound scans over a limited region of the muscle belly, assuming this represents the whole muscle, but we cannot confirm whether there were regional adaptations. Because we enrolled untrained participants, the present findings should be interpreted as likely reflecting early-phase adaptations to resistance training. In individuals with prior resistance-training experience, the magnitude and time course of neuromuscular and architectural adaptations may differ because of previous training exposure and a lower adaptive reserve. Future studies should address this, but the biomechanical differences observed here between the squat and leg-extension exercises, particularly with respect to the torque profile and the muscle lengths at which high contractile forces were produced, are likely to be relevant also in trained populations.

## Conclusion

Our data indicate that squat exercise is more effective than exercise at the leg extension machine used in the present study for inducing knee extensor muscle hypertrophy, neuromechanical adaptations, and a shift towards operation over longer fascicle lengths, but also for 1RM improvement transfer between the two exercises. These effects are likely due to the greater knee torque required to maintain a given external load (%1RM) in the squat, but also the longer length at which the knee extensors contract to produce the maximum force required to effectively perform this exercise.

## Supplementary Information

Below is the link to the electronic supplementary material.

Supplementary Material 1
