# Supplementary material for: Differential neuromuscular adaptations following multi-joint vs single-joint resistance exercise training
Source: Eur J Appl Physiol. 2026 Aug 8;126(11):6065–77. doi: 10.1007/s00421-026-06391-7 (PMC13751743; doi:10.1007/s00421-026-06391-7)
Supplement: Supplementary file 1 [file 421_2026_6391_MOESM1_ESM.docx]

**Differential neuromuscular adaptations following multi-joint vs single-joint resistance exercise training**

Andrea Monte^1,2^, Vasilios Baltzopoulos^2^, Paola Zamparo^1^, Dave Clarck^2^, Nicola Lucietto^1^, Constantinos N. Maganaris^2^

**Table S1:** training program. The program consisted of two days of training and a third session where the 1RM was evaluated to adapt to the intensity of the next week.

|  |  |  | ***First Day*** | |  | ***Second Day*** | |  | ***Third Day*** | |
| --- | --- | --- | --- | --- | --- | --- | --- | --- | --- | --- |
| ***Week 1*** | *SQG or LEG* |  | 5x10 | 60% 1RM |  | 3x7 | 65% 1RM |  | Test Day | |
|  |  |  |  |  |  | 3x5 | 70% 1RM |  |  |  |
|  |  |  |  |  |  |  |  |  |  |  |
| ***Week2*** | *SQG or LEG* |  | 1x4 | 75% 1RM |  | 3x3 | 80% 1RM |  | Test Day | |
|  |  |  | 1x3 | 80% 1RM |  |  |  |  |  |  |
|  |  |  | 1x3 | 83% 1RM |  | 3x2 | 85% 1RM |  |  |  |
|  |  |  | 2x4 | 80% 1RM |  |  |  |  |  |  |
|  |  |  |  |  |  |  |  |  |  |  |
| ***Week3*** | *SQG or LEG* |  | 1x4 | 80% 1RM |  | 2x3 | 80% 1RM |  | Test Day | |
|  |  |  | 2x3 | 83% 1RM |  | 3x3 | 87% 1RM |  |  |  |
|  |  |  | 3x2 | 87% 1RM |  | 2x3 | 83% 1RM |  |  |  |
|  |  |  | 2x3 | 83% 1RM |  |  |  |  |  |  |
|  |  |  |  |  |  |  |  |  |  |  |
| ***Week 4*** | *SQG or LEG* |  | 3x1 | 85% 1RM |  | 3x6 | 65% 1RM |  | Test Day | |
|  |  |  | 3x2 | 80% 1RM |  | 4x2 | 70% 1RM |  |  |  |
|  |  |  | 2x3 | 75% 1RM |  |  |  |  |  |  |
|  |  |  |  |  |  |  |  |  |  |  |
| ***Week 5*** | *SQG or LEG* |  | 5x4 | 80% 1RM |  | 5x3 | 85% 1RM |  | Test Day | |
|  |  |  |  |  |  |  |  |  |  |  |
| ***Week 6*** | *SQG or LEG* |  | 5x2 | 85% 1RM |  | 5x5 | 80% 1RM |  | Test Day | |
|  |  |  |  |  |  |  |  |  |  |  |
| ***Week 7*** | *SQG or LEG* |  | 6x4 | 80% 1RM |  | 1x2 | 80% 1RM |  | Test Day | |
|  |  |  |  |  |  | 2x2 | 85% 1RM |  |  |  |
|  |  |  |  |  |  | 2x2 | 90% 1RM |  |  |  |
|  |  |  |  |  |  | 2x2 | 80% 1RM |  |  |  |
|  |  |  |  |  |  |  |  |  |  |  |
| ***Week 8*** | *SQG or LEG* |  | 5x4 | 70% 1RM |  | 6x6 | 80% 1RM |  | Test Day | |
|  |  |  |  |  |  |  |  |  |  |  |
| ***Week 9*** | *SQG or LEG* |  | 1x2 | 80% 1RM |  | 4x4 | 80% 1RM |  | Test Day | |
|  |  |  | 1x1 | 85% 1RM |  |  |  |  |  |  |
|  |  |  | 1x1 | 90% 1RM |  |  |  |  |  |  |
|  |  |  | 1x2 | 92% 1RM |  |  |  |  |  |  |
|  |  |  | 2x2 | 80% 1RM |  |  |  |  |  |  |
|  |  |  |  |  |  |  |  |  |  |  |
| ***Week 10*** | *SQG or LEG* |  | 4x3 | 80% 1RM |  | 5x5 | 85% 1RM |  | Test Day | |

**Figure S1:** Muscle thickness and pennation angle individual data for all the investigated muscles. Red and blue dots represent PRE and POST training, respectively. Statistical analyses are reported in the main text
